# Influences of Trypsin Pretreatment on the Structures, Composition, and Functional Characteristics of Skin Gelatin of Tilapia, Grass Carp, and Sea Perch

**DOI:** 10.3390/md21080423

**Published:** 2023-07-25

**Authors:** Qiufeng Ruan, Weijie Chen, Min Lv, Rong Zhang, Xu Luo, Ermeng Yu, Chuanyan Pan, Huawei Ma

**Affiliations:** 1 Guangxi Key Laboratory of Aquatic Genetic Breeding and Healthy Aquaculture, Guangxi Academy of Fishery Sciences, Nanning 530021, China; r8682089@163.com (Q.R.); cwjmiss@yeah.net (W.C.); liaaa2021@126.com (M.L.); slimkent@126.com (X.L.); yem@prfri.ac.cn (E.Y.); 2 Liunan Modern Agricultural Service Center, Liuzhou 545007, China; zrpippo@163.com; 3 Guangdong Provincial Key Laboratory of Aquatic Animal Immunology and Sustainable Aquaculture, Pearl River Fisheries Research Institute of CAFS, Guangzhou 510380, China; 4 College of Food Science and Technology, Huazhong Agriculture University, Wuhan 430070, China

**Keywords:** trypsin, fish skin gelatin, structural properties, component, functional characteristics, HPLC-MS

## Abstract

Fish skin gelatin is an important functional product in the food, cosmetics, and biomedicine industries, and establishing a green and effective fish skin gelatin extraction method is an effective way to obtain high-quality gelatin and improve its production efficiency. In this study, a trypsin method was used to extract the skin gelatin of sea perch, tilapia, and grass carp, and the microstructures of skin gelatin of these three fish species were analyzed, with such functional characteristics as thermal stability, gel strength, and emulsifying properties measured. The study results show that the skin gelatin of sea perch and tilapia obtained through the trypsin method has a relatively big molecular mass, a dense network structure, and a stable trihelix conformation. In addition, the skin gelatin of these three fish species has a relatively high β-turn content in the secondary structure, good gel strength, and water absorption properties. The compositions of the collagen-associated proteins in the skin gelatins of these three fish species extracted with the trypsin method are significantly different from each other, with positive effects of decorin and biglycan on the stability of the network structure of gelatin and a certain damaging effect of metalloendopeptidase on the network structure of gelatin. The skin gelatin of tilapia has high thermal stability and good emulsifying performance. Therefore, this gelatin type has bright application prospects in such fields as food processing, cosmetics, and drug development. In contrast, the skin gelatin of grass carp has poor functional properties. Therefore, there are significant differences among the structures and functions of skin gelatin extracted from different kinds of fish through the trypsin method. This finding has provided a useful reference for the production of customized fish gelatin according to demand.

## 1. Introduction

Gelatin is a general term for the main product of collagen after mild and irreversible breakage and results from the partial denaturation of collagen, the main structural protein in connective tissue. It is widely used in various industries such as food, pharmacy, cosmetics, and photography. Gelatin mainly comes from pork, cowhide, and the bones of swine and cows. In recent years, the production volume of gelatin from other raw materials has significantly increased. These raw materials include the bones, cartilage, and skins of fish [1,2,3]. There are two primary reasons why people are interested in fish gelatin: On the one hand, using by-products in the fishery industry to produce gelatin can not only utilize the fishery waste in a reasonable way and reduce the environmental pollution but also effectively improve the fishery economy. On the other hand, the raw material types and chemical composition of gelatin are important factors influencing the properties of gelatin [4,5]. Fishery by-products (such as skin, scales, bones, fins) have a high content of collagen, abundant raw materials, and low prices, and they are rare high-quality protein resources and important raw materials for producing high-quality gelatin.

The extraction methods and extraction conditions (such as pH value, temperature, and time) of gelatin can determine the yields and functional properties of gelatin produced. Traditional methods of gelatin extraction include thermal extraction, acid extraction, and alkali extraction. The gel strength and viscosity of gelatin obtained through thermal extraction are relatively low. Meanwhile, the methods of acid extraction and alkali extraction have the problems of long processing time of raw materials, environmental pollution, low gelatin viscosity and freezing strength, and poor gelatin quality [6]. With the development of biotechnology during these years, using the enzymatic method in extracting gelatin has been favored by researchers. With the advantages of short extraction time, high gelatin yield, stable physical and chemical properties of gelatin, and small pressure on the environment, the enzymatic extraction method has become a green and effective method for gelatin extraction. In the production of fish gelatin, a mild and economical extraction method, a relatively short extraction time, and a weak acid environment for producing high-quality gelatin are required [7]. Using enzymes to extract gelatin from fish materials is one of the optimal methods for improving the production processes of fish gelatin. Due to their particularity, enzymes have a targeting effect on some molecular bonds of collagen and can improve the digestion rates of raw materials. Generally, natural enzymes are used in gelatin extraction, including papain from plants and pepsin and trypsin from animals. Li et al. used the enzymolysis method to extract gelatin from swine skins, with the complete trihelix structure well preserved [8]. Masahiro et al. used the enzymolysis method to extract gelatin from the skins of black drum and mutton snappers, with extraction rates of 44.7% and 35%, respectively [9]. Using the enzymolysis method, Jongjareonrak also extracted gelatin from vermilion snappers [10]. Tilapia, grass carp, and Sea brass are important economic fishery resources in China, with 60–70% of their bodies used as by-products. Against the backdrop of high aquaculture yields, the disposal of aquaculture by-products (such as heads, bones, skins, scales, and viscera) has become an urgent problem to be resolved [11]. Fish skins are a primary by-product of the fishery and aquaculture industry. They contain a large amount of collagen, with a content percentage of up to 90%. Therefore, they are precious raw materials for gelatin production [7,12]. Extracting gelatin from fish skins is a good way to effectively use the waste of the aquaculture industry, increase its added value, and obtain high-quality gelatin products.

In the production of gelatin from aquaculture by-products, the improvements of production technology are crucial for maintaining the physicochemical and functional properties of gelatin products, improving the gelatin yields, shortening the production time, and reducing the production costs. In this study, we have extracted gelatin from skins of sea perch, tilapia, and grass carp using trypsin, observed the characteristics of micro-structures and trihelix structures of skin gelatin of these three fishes, analyzed the functional properties of gelatin, and further explored the interactions among gelatin components. Therefore, this study has provided a reference for the production and application study of customized gelatin.

## 2. Results

### 2.1. Gelatin Molecular Mass

**Figure 1:**
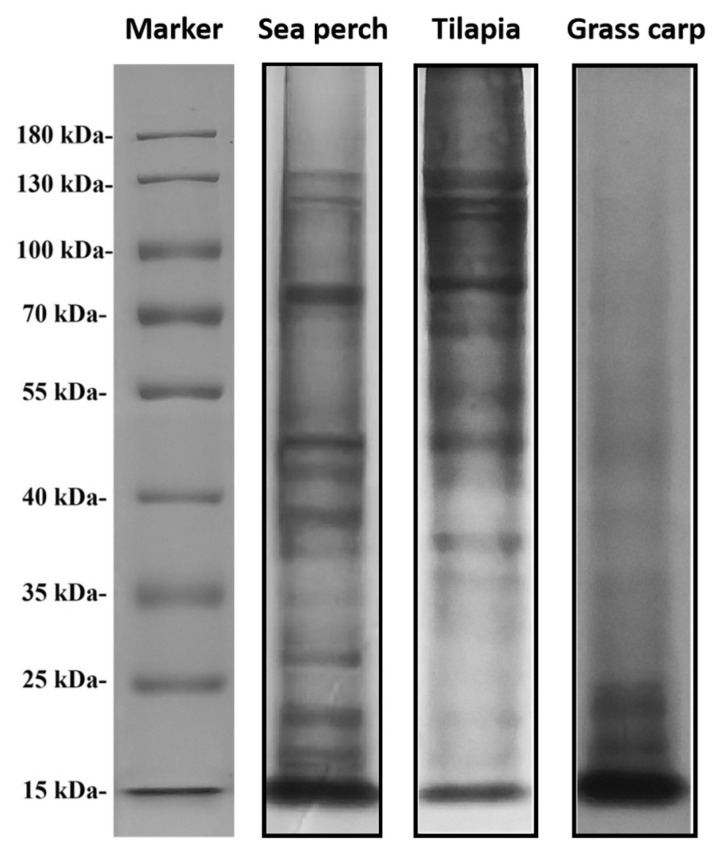
SDS-PAGE patterns of skin gelatins extracted from sea perch, tilapia, and grass carp with a trypsin method.

Molecular size is one important parameter determining the properties of gelatin. In this study, the molecular mass of gelatin was observed with the sodium dodecyl sulfate-polyacrylamide gel electrophoresis (SDS-PAGE), as shown in Figure 1. The skin gelatin extracted from grass carp through the trypsin method only shows bands of low molecular masses, with a range from 15 kDa to 25 kDa. In contrast, the skin gelatin extracted from sea perch and tilapia shows bands of different molecular masses, with a band of the largest molecular mass at 120 kDa and a band of the smallest molecular mass at 15 kDa. The study results show that trypsin has a more effective enzymolysis effect on the skins of grass carp, with their collagenases decomposed into small molecular fragments.

### 2.2. SEM Analysis

**Figure 2:**
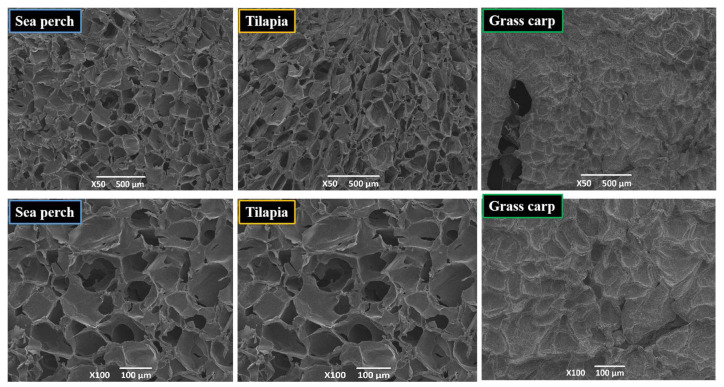
Scanning electron microscope (SEM) observation results of skin gelatins extracted from sea perch, tilapia, and grass carp with a trypsin method.

Figure 2 shows the micro-structures of skin gelatin of sea perch, tilapia, and grass carp observed with a scanning electron microscope. With a magnification of 50, the microscope shows a clear and spongy porous mesh structure of the skin gelatin of sea perch and tilapia, with evenly distributed and densely arranged mesh pores. Meanwhile, the skin gelatin of grass carp has no apparent mesh structure and mesh pores. In the skin gelatin of grass carp, there are irregular shallow pits, indicating a certain depolymerization and damaging effect of trypsin on the skin gelatin molecules of grass carp. With a magnification of 100, the microscope shows that the netting of the mesh structure of the skin gelatin of sea perch is upright, while the netting of the mesh structure of the skin gelatin of tilapia is slanted, with some collapsed mesh pores. Trypsin has the strongest damaging effect on the skin gelatin molecules of grass carp and the second strongest damaging effect on the skin gelatin molecules of tilapia. The damaging effect of trypsin on the skin gelatin molecules of sea perch is the slightest.

### 2.3. FT-IR and UV-Vis Characterization

**Figure 3:**
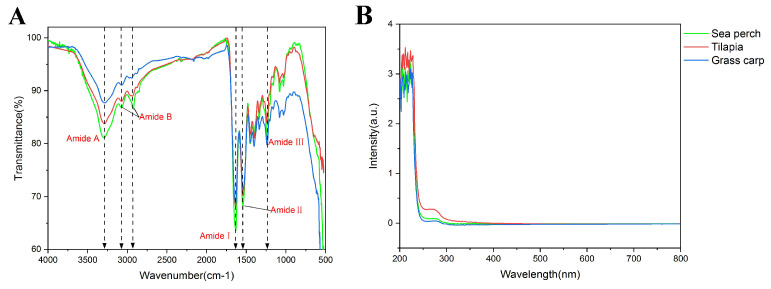
FT-IR (**A**) and UV-vis (**B**) spectra of skin gelatin extracted from sea perch, tilapia, and grass carp with the trypsin method.

Skin gelatin extracted from sea perch, tilapia, and grass carp with the trypsin method shows the infrared spectral characteristic absorption peaks of collagen (Amide A, B, I, II, and III), as presented in Figure 3A. The Amide A absorption peaks of skin gelatin all appear around 3290 cm^−1^, with the Amide A absorption peaks of skin gelatin of sea perch and tilapia appearing at 3293 cm^−1^ and the Amide A absorption peaks of skin gelatin of grass carp appearing at 3287 cm^−1^. The sequence in terms of the Amide A band absorption intensity (from the highest to the lowest) is as follows: sea perch, tilapia, and grass carp. The Amide I band absorption peaks of these three kinds of gelatin all appear around 1632 cm^−1^, with the Amide I band absorption peak of skin gelatin of tilapia appearing at 1634 cm^−1^ and the Amide I band absorption peaks of skin gelatin of grass carp and sea perch appearing at 1633 cm^−1^ and 1632 cm^−1^, respectively. The skin gelatin of sea perch has the highest Amide I band absorption intensity, followed by the skin gelatin of tilapia and grass carp with similar absorption intensities. The ultraviolet absorption spectra of skin gelatin of tilapia, sea perch, and grass carp extracted with the trypsin method are similar (Figure 3B), with their maximum absorption peaks appearing at the wavelengths of 212 nm, 222 nm, and 222 nm, respectively. Meanwhile, a red-shift phenomenon appears at their maximum absorption wavelengths, and the intensities of their ultraviolet absorption peaks vary. The sequence from the highest to the lowest in terms of the ultraviolet absorption peak intensity is the skin gelatin of tilapia (3.53), the skin gelatin of sea perch (3.25), and the skin gelatin of grass carp (3.10). This result indicates that there are some differences among the skin gelatin structures of these three fish species.

### 2.4. Gelatin Secondary Structure (CD)

**Figure 4:**
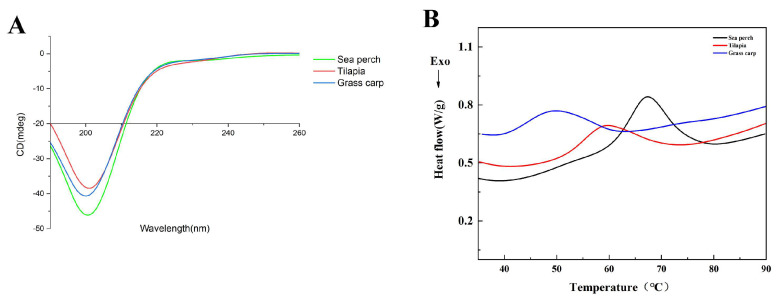
CD spectra (**A**) and thermostability (**B**) of skin gelatin extracted from sea perch, tilapia, and grass carp with the trypsin method.

In this study, CD spectra were used to investigate the changes in the secondary structures of skin gelatin of different fish species. Figure 4A shows that, from the spectra of skin gelatin of sea perch, tilapia, and grass carp extracted with the trypsin method, it can be seen that there are negative peaks appearing at the wavelength of 200 nm with different peak intensities and that these negative peaks remain within a wavelength range of 220–230 nm. It indicates that the secondary structures of skin gelatin of sea perch, tilapia, and grass carp are primarily random coil structures. Meanwhile, the secondary structures of these three kinds of fish skin gelatin have different contents, with the random coil ratios of skin gelatin of sea perch, tilapia, and grass carp at 61.5%, 61.1%, and 61.2%, respectively, and the β-turn ratios of these three kinds of fish gelatin at 23.8%, 25.3%, and 18.7%, respectively.

### 2.5. DSC Analysis

Figure 4B shows that during the heating process, all these three kinds of fish skin gelatin obtained with the trypsin method present a heat absorption peak, with the absorption peaks of the skin gelatin of sea perch, tilapia, and grass carp appearing at 67 °C, 59 °C, and 49 °C, respectively. It indicates that the skin gelatin of sea perch has the highest thermal stability, followed by the skin gelatin of tilapia. The thermal stability of the skin gelatin of grass carp is the worst.

### 2.6. HPLC-MS Analysis

**Figure 5:**
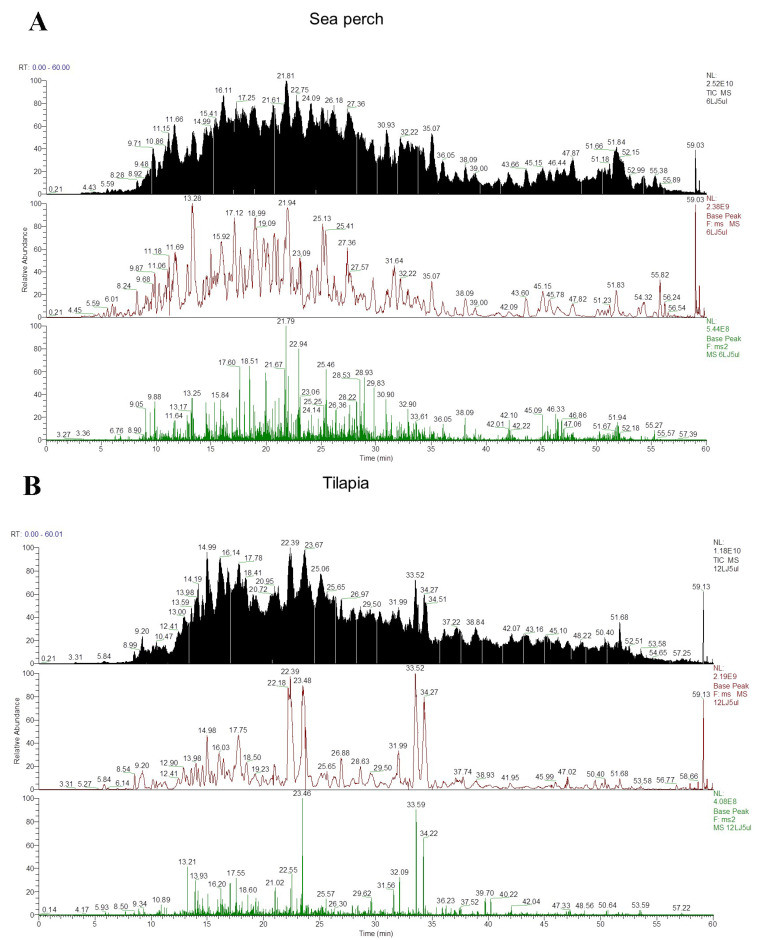

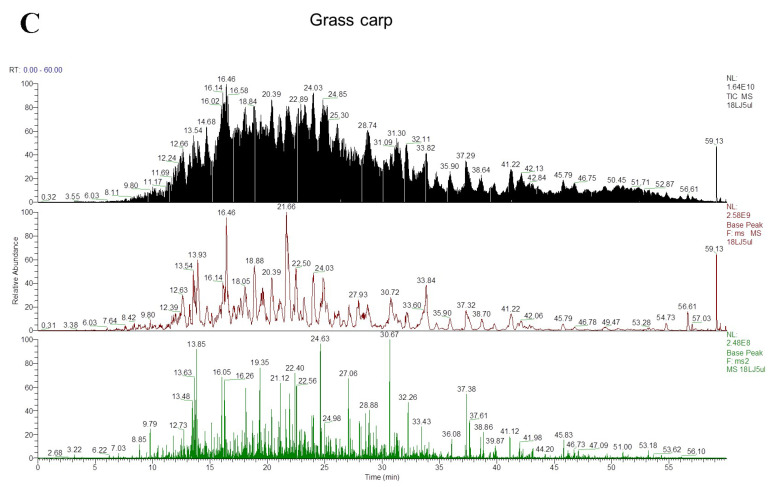

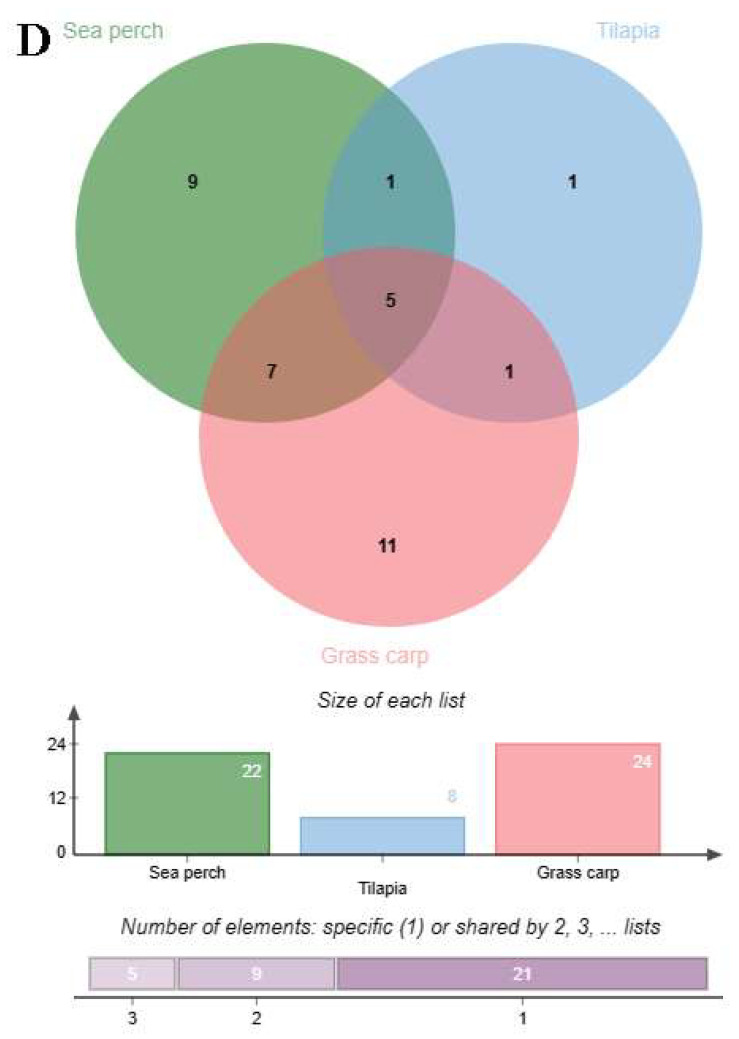
Total ion chromatograms of three fish skin (sea perch, tilapia, and grass carp skin extracted by trypsin methods) gelatin measured with high-performance liquid chromatography-mass spectrometry (HPLC-MS) (**A**–**C**) and Venn diagram of the composition of collagen-associated proteins (**D**).

In this study, the protein composition of these three kinds of fish skin gelatin was analyzed with the HPLC-MS measurement method and a reference to the sequence database of zebrafish collagen (as shown in Figure 5). Table S1 shows the measurement and identification results of the fish skin gelatin collagen and collagen-associated proteins. The identification result shows that there are twenty-two, eight, and twenty-four types of collagen and collagen-associated proteins in the skin gelatin of sea perch, tilapia, and grass carp, respectively. In addition, these three kinds of fish skin gelatin contain collagen types I, II, VI, and XIV. The composition of the collagen-associated proteins in these three kinds of fish skin gelatin extracted with the trypsin method is significantly different. Five types of collagen-associated proteins were measured in the skin gelatin of sea perch. These protein types include the decorin protein, Col2a1a protein, lumican (Fragment), Proline/arginine-rich end leucine-rich repeat protein, and collagen and calcium-binding EGF domain-containing protein 1. Only one collagen-associated protein type, biglycan, was measured in the skin gelatin of tilapia, and four collagen-associated protein types, namely, Col2a1a protein, Si:ch211-106n13.3, Si:ch211-157b11.8, and metalloendopeptidase, were measured in the skin gelatin of grass carp.

### 2.7. Analysis of Gel Properties

**Figure 6:**
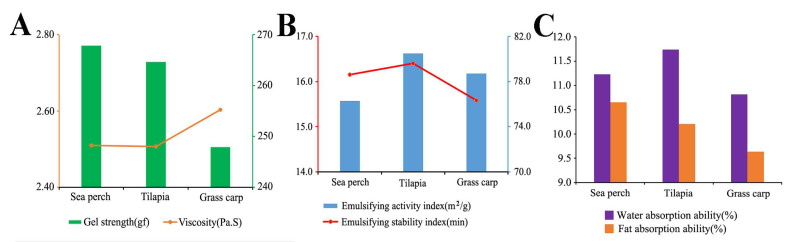
Functional properties: gel strength (**A**); emulsifying properties (**B**); lipid absorption performance (**C**) of skin gelatin extracted from sea perch, tilapia, and grass carp with the trypsin method.

As shown in Figure 6A, the gel strength of skin gelatin of sea perch and tilapia is relatively high (2.63 ± 0.06 N and 2.59 ± 0.08 N), and the gel strength of skin gelatin of grass carp is relatively low (2.43 ± 0.08 N). In addition, there are no significant differences in the viscosity of these three fish skin gelatin types. The emulsifying properties and stability of the skin gelatin of tilapia are relatively good (Figure 6B). Compared with the skin gelatin of grass carp, the skin gelatin of tilapia and sea perch has a better water and lipid absorption performance (Figure 6C).

## 3. Discussion

### 3.1. Structures of Gelatin and Its Relation to Functional Properties

In this study, the skin gelatin of sea perch, tilapia, and grass carp was extracted with the trypsin method, and then the structural and functional properties of these three gelatin types were analyzed. The study results show that the skin gelatin of these three fish species extracted with the trypsin method has different network structures, secondary structures, and spatial conformations and that these differences lead to different functional properties among these three gelatin types. The functional properties of gelatin are closely related to its network structure. The gel microstructure of fish skin gelatin exhibits uniform sizes of surface pores, which are well cross-linked and densely arranged, indicating good formation of its gel structure and great gel strength [13]. Some research has found that gels with a finer and denser network structure have enhanced water absorption properties (such as water-retaining capability, water absorption, and so on) [14]. This study has shown that the skin gelatin of sea perch and tilapia has a spongy and porous mesh structure with densely arranged mesh pores, and these two gelatin types have good gel strength and water and lipid absorption performance. These findings have further verified the research result described above. An explanation for these findings is that under the same extraction conditions, trypsin could have a relatively low enzymolysis effect on the skin collagen of sea perch and tilapia. Meanwhile, with a strong binding capability with the skin collagen of grass carp, these trypsins have a more effective enzymolysis effect on the skin collagen of grass carp, thus damaging the skin gelatin structure of this fish species.

The secondary structure and spatial conformation of gelatin also affect its functional properties. Gelatin with a high β-turn content and a stable trihelix conformation shows high gel strength and good water absorption performance. The gel strength of gelatin is related to the β-turns of its secondary structure. An increase in β-turns is conducive to the formation of a more uniform and finer network of gelatin, thus enhancing its gel strength [15]. This study has shown that the skin gelatin of sea perch and tilapia extracted with the trypsin method has relatively good water and lipid absorption properties, a high content of β-turn, and a more uniform and finer network structure. A possible reason is that trypsins have a strong ability to hydrolyze collagen molecules, which increases the number of β-turns of gelatin and forms a more uniform and finer network structure, thus enhancing the water absorption properties of gelatin [16]. In the future, this method can be used to extract fish skin gelatin with good water and lipid absorption properties for food (such as margarine and salad dressings) and cosmetics (such as creams and cleansing oil) applications [17].

In addition, some research has found that the swine-skin gelatin extracted with trypsin has a fine and dense network structure and a stable trihelix conformation, as well as good water and lipid absorption properties [18]. In this study, it has been found that the skin gelatin of sea perch and tilapia extracted with trypsin has a stable trihelix conformation and good water and lipid absorption properties. Thus, this finding has further verified the study results of other scholars. The skin gelatin of sea perch has the biggest Amide I band amplitude, which indicates a most stable trihelix conformation of this gelatin type. It could be because sea perch lives in the ocean, where the surrounding saline solution has enhanced the network structure stability of its skin gelatin through the electrostatic interactions among molecules. Therefore, the trihelix conformation of this gelatin type is not easily disrupted.

### 3.2. Component of Gelatin and Its Relation to Functional Properties

Gelatin with an ordered internal structure usually has excellent thermal stability. The thermal denaturation of protein can be reflected by a dynamic change in temperature. With the increase of heating temperature, energy is absorbed, and hydrogen bonds in gelatin molecules break, resulting in the change of the state of gelatin structure from an ordered one to a disordered one and the unfolding of protein molecules [19]. Gelatin is a hydrolysis product of collagen, and the thermal stability and internal structure state of gelatin can be judged by its heat absorption peak. In this study, it has been found that the skin gelatin of sea perch and tilapia has relatively high thermal stability, while the thermal stability of the skin gelatin of grass carp is relatively low. This result has indicated that the skin gelatin of sea perch and tilapia has ordered and stable gel network structures. It is consistent with the result obtained with a scanning electron microscope in this study. The microstructure of gelatin determines its gel strength, and gelatin with higher gel strength has better mechanical and barrier properties [6]. In this study, high gel strength was measured in the skin gelatin of sea perch and tilapia. One possible reason is that under these extraction conditions, trypsin has a less effective enzymolysis effect on the skin collagen of sea perch and tilapia, resulting in the more compact and more stable gel network structures and the enhanced gel strength of these two gelatin types [20]. Therefore, these gelatin types have a bright application prospect in such fields as food packaging, cosmetics, and pharmaceutical packaging.

When collagen-associated proteins in gelatin such as decorin, biglycan, and collagen and calcium-binding EGF domain-containing protein 1 cross link with the collagen, the structural and functional properties of gelatin will be affected [21]. Decorin can perform the non-covalent cross-linking with most collagen types (I, II, IV, V, VI, VII, XI, XII, XIV, and XXI), regulate the forming rates and degrees of collagenous fibers [22], and participate in the sequencing of collagenous fibers, thus resulting in a relatively dense network structure and enhanced thermal stability of fish skin gelatin [23]. The skin gelatin of sea perch investigated in this study contains decorin. In addition, this gelatin type has a relatively dense network structure and high thermal stability. Therefore, it has been proven that decorin has performed the non-covalent cross-linking with collagen types I, II, IV, V, and VI in the skin gelatin of sea perch, with gelatin with good thermal stability and a dense network structure generated. It has been found in this study that biglycan can cross link with collagen types I and II, and this finding can be used to transform the morphology of collagen fibers into a fine and dense network, thus improving the gel strength and viscosity of gelatin to produce better biological coating and scaffold materials [24]. This study has also found that the skin gelatin of tilapia containing biglycan has better gel strength and viscosity and a denser network structure, indicating that biglycan can improve the network structure and structural characteristics of the skin gelatin of tilapia. However, metalloendopeptidase can bind with collagen and split triple helix peptides, resulting in a damaged network structure of gelatin [25]. In this study, metalloendopeptidase was only measured in the skin gelatin of grass carp. Combining it with the results obtained with the scanning electron microscope, we have found that the skin gelatin of grass carp has a porous and non-dense network structure with non-uniform pore sizes, which indicates that metalloendopeptidase has a certain damaging effect on the network structure of fish skin gelatin. There are significant differences in the collagen composition of skin gelatin of those three fish species extracted with trypsin. In addition, their structural and functional properties vary significantly. The speculation is that the differences among the skin structures of these three fish species result from their different living environments. The emulsifying properties of gelatin are closely related to the lengths of peptide chains. Small molecule short-chain peptides can quickly migrate to the gelatin surface and diffuse around oil droplets, thus increasing the area of contact surface [26]. The skin gelatin of tilapia and grass carp extracted with trypsin in this study has good emulsifying properties. One possible reason is that trypsin has split the collagen into more short-chain peptides containing more low-molecular-weight fragments [27,28], which provide more electriferous groups at the ends of polypeptides and increase the area of the contact surface, thus improving the emulsifying properties of gelatin. Fish skin gelatin prepared with this method can be used as a foaming agent or an emulsifying agent in such application fields as food, pharmacy, and medical technology [29].

## 4. Materials and Methods

### 4.1. Materials and Reagents

Skins (tilapia, grass carp, and sea perch) were provided by Guangxi Luchi Agriculture Co., Ltd. (Nanning, China); fresh fish skins were frozen under −18 °C, and then transported to the laboratory in refrigerated trucks. The fish skin was manually stripped of scales and muscle fragments using a knife, thoroughly washed in tap water, frozen, and stored at −20 °C before use. Other materials used include trypsin (250 U/mg) (Shanghai Macklin Biochemical Technology Co., Ltd., Shanghai, China), 10% SurePAGE Bis-Tris gels (GenScript, Nanjing, China), and SpectraTM Broad Multicolor High Range Protein Ladder Standard (10–180 kDa, GenScript, Nanjing, China).

### 4.2. Gelatin Extraction

The frozen skins were then defrosted at room temperature, washed with tap water, and used to extract the gelatin. A total of 200 g of fresh fish skins were washed and then soaked in 500 mL 0.025 mol/L sodium hydroxide solution for 12 h to remove any non-collagenous proteins. After being well cleaned with distilled water, a total of 500 mL of 0.01 mol/L glacial acetic acid was added to swell fish skin for 3 h. A total of 400 g of fish skin was weighed for extraction using trypsin methods after being washed. A volume of 500 mL of water and a weight of 0.3 g of trypsin were added to the pretreated skins. The pH value of the solution was adjusted to 8.1 with the 1 mol/L sodium hydroxide solution, and the solution was homogenized after a water bath at 37 °C for 20 min. The homogenate was boiled for 30 s to inactivate the enzyme, and mogenate was agitated in a water bath set at 45 °C overnight before the gelatin solution was filtered through four layers of gauze. The resultant liquid was centrifuged at 6000 rpm for 20 min at room temperature to obtain gelatin. Finally, dried gelatins were obtained by vacuum freeze-drying all gelatin solutions using a freeze dryer (2KBTES-55, VirTis Co., Gardiner, NY, USA).

### 4.3. SDS-PAGE

A total of 0.02 g of gelatin sample was pretreated according to the method introduced by Zhang et al. (2020) [6]. Then, a volume of 10 μL of gelatin sample was loaded onto 10% SurePAGE Bis-Tris gels (GenScript, Nanjing, China). The applied electrophoresis voltage and electrophoresis time were 120 V and 80 min, respectively. The SpectraTM Broad Multicolor High Range Protein Ladder Standard (10–180 kDa, GenScript, Nanjing, China) was applied in this study as the protein standard. The electrophoresis gel was treated with a staining solution (1 g/L Coomassie Brilliant Blue R-250, 0.25 g/mL isopropanol and 0.1 g/mL acetic acid) for 2 h, and it was then treated with a destaining solution (0.2 g/mL ethanol and 0.1 g/mL acetic acid). When clear bands were observed, the gel was taken out and photographed with a digital camera.

### 4.4. Scanning Electron Microscope (SEM) Observation

The gelatin samples were freeze-dried for 48 h, generating the lyophilized gelatin samples. Then, these samples were sliced into 5 mm × 5 mm × 1.5 mm slices. After they were glued to the sample stage, the slices were sputter coated with gold. Then, they were observed with a BIOSEM (JSM-6390LV, NTC, Tokyo, Japan) at an accelerating voltage of 10 kV.

### 4.5. UV-Vis and FT-IR Spectroscopy

The UV-vis spectra of the gelatin samples at wavelengths of 200–800 nm were recorded with a SpectraMax i3x UV-visible spectrophotometer (Molecular Devices, San Jose, CA, USA). A total of 10 mg of the lyophilized samples was fixed with ATR cells. Then, they were scanned 32 times with an FTIR (Nicolet 6700, Thermo Scientific, Waltham, MA, USA) within the wavenumber range of 4000–500 cm^−1^, under a spectral resolution of 2 cm^−1^.

### 4.6. Circular Dichroism (CD) Spectra Assay

Some 0.5 mol/L acetic acid was added to 1 mg of the gelatin sample. Then, the sample was used to prepare a collagen solution with a concentration of 0.1 mg/mL. The prepared collagen solution was centrifuged at 10,000 rpm for 30 min at 4 °C to remove impurities. After that, the solution was injected into a quartz cuvette (with a 1 mm light path) to 2/3 of its height, with bubbles avoided. With the 0.5 mol/L acetic acid as a background solution, a CD spectrometer (J-1500, JASCO, Tokyo, Japan) was applied to analyze the gelatin sample under the following conditions: a temperature of 25 °C, a spectral scan range of 190–260 nm, a step interval of 0.1 nm, a response time of 1 s, and a data acquisition mode of spectrum. In order to measure the sample spectra at different wavelengths, the following different nitrogen flow rates were applied: 2–4 L/min (190–400 nm); 3–5 L/min (185 nm); 10 L/min (180 nm); 30–50 L/min (175 nm); and 60–70 L/min (170 nm), with the absorption of ultraviolet light by ozone prevented from interfering in the spectra measured.

### 4.7. Differential Scanning Calorimetry (DSC) Assay

A total of 5.0 mg of the gelatin sample was weighed precisely. Then, the sample was placed into a DSC aluminum crucible. After that, the crucible was sealed with a tablet press. Meanwhile, an empty crucible was placed in the sample cell as a blank control. After that, these two crucibles were heated in a DSC (DSC 204 F1, NETZSCH, Munich, Germany). The initial heating temperature was set as 30 °C, and the heating rate was set to 10 °C/min. These two crucibles were then heated until the heating temperature reached 120 °C. Then, the gelatin sample was scanned, with its spectral data analyzed with the thermal analysis software of the instrument.

### 4.8. High-Performance Liquid Chromatography-Mass Spectrometry (HPLC-MS) Assay

A total of 50 μg of the gelatin sample was diluted in 100 μL of 50 mmol/L ammonium bicarbonate. Then, a volume of 25 μL of 6 mol/L guanidine hydrochloride was added in the diluent. After that, DTT with a concentration of 2 mmol/L was added in the diluent. Then, the diluent was heated at 60 °C for 30 min. After that, the diluent was cooled down to room temperature. An iodoacetamide solution with a concentration of 5 mmol/L was added in the diluent. Then, the mixture was placed in darkness for reaction for 30 min. After that, more DTT with a concentration of 2 mmol/L was added in the diluent. Then, it was incubated with a centrifugal speed of 700 rpm for 10 min at the room temperature. A certain volume of trypsin solution was added in the test tube, and the mixture was incubated at 37 °C for 16 h. After that, some 0.1 g/mL TFA was added to the mixture to stop its chemical reaction. Then, the sample was processed in a centrifugal concentrator (Thermo Mixer, Eppendorf, Hamburg, Germany). Desalting was performed with the method introduced by Lueyot et al. [30], and the sample was then analyzed with high-performance liquid chromatography (EASY nLC 1200, Thermo Fisher Scientific, Waltham, MA, USA) and the mass spectrometer (Q Exactive Plus, ThermoFisher Scientific, Waltham, MA, USA). A chromatographic column (Pepmap RSLC, C18, 2 μm, 100 Å, 50 μm × 15 cm) and the mobile phases (A: 1.22 g/L formic acid; B: 621 g/L acetonitrile/1.22 g/L formic acid) were applied to analyze the sample with a flow rate of 300 nL/min. The following gradients were used in LC separation: 3–10% B for 10 min; 10–28% B for 35 min; 28–38% B for 8 min; and 38–100% B for 7 min. With a reference database of zebrafish collagen sequence, the software Proteome Discoverer 2.2 (Thermo Fisher Scientific, Waltham, MA, USA) was used to search the raw mass spectral data of the samples.

### 4.9. Determination of Gelatin Properties

The gel strength of the gelatin sample was measured with the method introduced by Rodsuwan et al. [31]. However, the method was modified slightly as follows: A texture analyzer (TA. newplus, iSENSO, New York, NY, USA) with a P/0.5 probe was applied to perform the breaking test, with the speed before the test being 1.0 mm/s, the speed during the test being 1.0 m/s, and the speed after the test being 5.0 mm/s. The measured deformation of the gelatin sample was 4 mm, and the measured induction force of the sample was 0.049 N. A viscosity measurement at the room temperature with a unit of Pa·s was performed on the gelatin solution. In addition, the emulsifying capacity and emulsion stability of the gelatin sample were measured with the method introduced by Wang and Kinsella [32]. In order to perform the measurement, a total of 10 g of gelatin sample was dissolved and bathed in water at 60 °C. Then, the emulsifying capacity and emulsion stability of the gelatin collected were identified. The water absorption capacity (WAC) and fat absorption capacity (FAC) of the gelatin sample were identified with the method introduced by Park and Kim [26]. A total of 10 g of the gelatin sample was transferred into each of two 50 mL centrifuge tubes filled with 10 g of pure water and 10 g of pure palm oil separately to identify the WAC and FAC of the sample.

### 4.10. Data Analysis

The results are presented as the mean ± standard deviation after all data were averaged in triplicates. The one-way analysis of variance module of the Statistics 9.0 program was used to analyze the significant differences (*p* < 0.05) among the data. Origin 2018 was used to perform the mapping.

## 5. Conclusions

In this study, the skin gelatin of sea perch, tilapia, and grass carp was extracted with the trypsin method, with its structural and functional properties analyzed. The study results show that there are significant differences among the structural and functional properties of these three fish skin gelatin types. In addition, the skin gelatin of sea perch and tilapia extracted with the trypsin method has a relatively large molecular mass, dense network structure, and stable trihelix conformation. Meanwhile, these two gelatin types have relatively high β-turn contents in their secondary structures, good gel strength, and good water absorption and emulsifying properties. This study has presented the structural and functional properties of fish skin gelatin obtained with the trypsin method, thus providing a useful reference for the targeted development of gelatin products. In the future, we will optimize the extraction conditions of the trypsin extraction method to improve the yield of gelatin and explore the applications of gelatin in different industries.

## Data Availability

Data are contained within the article or Supplementary Material.

## Supplementary Materials

The following supporting information can be downloaded at https://www.mdpi.com/article/10.3390/md21080423/s1: Table S1: The identification results of the fish skin gelatin collagen and collagen-associated proteins by High performance liquid chromatography-mass spectrometry (HPLC-MS).
