# Supplementary material for: Influences of Trypsin Pretreatment on the Structures, Composition, and Functional Characteristics of Skin Gelatin of Tilapia, Grass Carp, and Sea Perch
Source: Mar Drugs. 2023 Jul 25;21(8):423. doi: 10.3390/md21080423 (PMC10456007; doi:10.3390/md21080423)
Supplement: Supplementary file 1 [file marinedrugs-21-00423-s001.zip › marinedrugs-2467474-supplementary.pdf]

Supplementary Table

Table S1 The identification results of the fish skin gelatin collagen and collagen-associated proteins by High performance liquid chromatography-mass spectrometry (HPLC-MS)

Items	Sample		
	Sea perch skin gelatin	Tilapia skin gelatin	Grass carp skin gelatin
Collagen types	Collagen, type I, alpha 1a	Collagen alpha chain	Collagen alpha chain
	Collagen, type I, alpha 1b	Collagen, type I, alpha 1b	Procollagen type I alpha 2 chain
	Collagen, type I, alpha 2	Collagen type II, alpha-1b	Collagen, type I, alpha 1a
	Collagen, type II, alpha 1b	Collagen, type VI, alpha 1	Collagen, type I, alpha 1b
	Collagen type II, alpha-1b	Collagen, type VI, alpha 2	Collagen type II, alpha-1b
	Collagen, type IV, alpha 5 (Alport syndrome)	Collagen, type XII, alpha 1a	Collagen, type II, alpha 1b
	Procollagen, type V, alpha 1 (Fragment)	Collagen, type XIV, alpha 1a	Collagen, type IV, alpha 6
	Collagen, type V, alpha 2a (Fragment)	Biglycan (Fragment)	Procollagen, type V, alpha 1 (Fragment)
	Collagen, type VI, alpha 1		Collagen, type V, alpha 2b
	Collagen, type VI, alpha 2		Collagen, type V, alpha 3a
	Collagen, type VI, alpha 3 (Fragment)		Collagen, type VI, alpha 1
	Collagen, type VI, alpha 3		Collagen, type VI, alpha 2
	Collagen, type XII, alpha 1a		Collagen, type VI, alpha 3 (Fragment)
	Collagen, type XII, alpha 1b		Collagen, type VI, alpha 3
	Collagen, type XIV, alpha 1a		Collagen, type VII, alpha 1
	Collagen type XV-B alpha 1 chain		Collagen type XI alpha 2 (Fragment)
	Collagen type XXVIII alpha 2 a		Collagen, type XII, alpha 1b
	Decorin		Collagen, type XIV, alpha 1a
	Col2a1a protein		Collagen, type XXI, alpha 1
	Lumican (Fragment)		Collagen alpha-1(XXVII) chain B
	Proline/arginine-rich end leucine-rich repeat protein		Col2a1a protein
	Collagen and calcium-binding EGF domain-containing protein 1		Si:ch211-106n13.3
			Si:ch211-157b11.8
			Metalloendopeptidase
